# Rapid Naming in Brazilian Students with Dyslexia and Attention Deficit Hyperactivity Disorder

**DOI:** 10.3389/fpsyg.2016.00021

**Published:** 2016-01-26

**Authors:** Luciana Mendonça Alves, Cláudia M. Siqueira, Maria do Carmo Mangelli Ferreira, Juliana Flores Mendonça Alves, Débora F. Lodi, Lorena Bicalho, Letícia C. Celeste

**Affiliations:** ^1^Department of Speech and Language Therapy, Centro Universitário Metodista Izabela Hendrix (Izabela Hendrix University)Belo Horizonte, Brazil; ^2^Laboratório de Estudo dos Transtornos de Aprendizagem – LETRA (Learning Disorders Study Laboratory) of Hospital das Clínicas, Universidade Federal de Minas GeraisBelo Horizonte, Brazil; ^3^Department of PediatricsFederal University of Minas Gerais, Brazil; ^4^Department of Speech, Language and Hearing ScienceUniversity of Brasília, Brazil

**Keywords:** dyslexia, ADHD, cognition, evaluation, rapid automatized naming

## Abstract

**Introduction:** The effective development of reading and writing skills requires the concerted action of several abilities, one of which is phonological processing. One of the main components of phonological processing is rapid automatized naming (RAN)—the ability to identify and recognize a given item by the activation and concomitant articulation of its name.

**Objective:** To assess the RAN performance of schoolchildren with dyslexia and attention deficit hyperactivity disorder (ADHD) compared with their peers.

**Methods:** In total, 70 schoolchildren aged between 8 and 11 years participated in the study. Of these, 16 children had a multiprofessional diagnosis of ADHD while 14 were diagnosed with dyslexia. Matched with these groups, 40 schoolchildren with no history of developmental impairments were also evaluated. The RAN test was administered to assess the length of time required to name a series of familiar visual stimuli. The statistical analysis was conducted using measures of descriptive statistics and the 2-sample *t*-test at the 5% significance level.

**Results:** The performance of the group with dyslexia was inferior to that of the control group in all tasks and the ADHD group had inferior performance for color and letters-naming tasks. The schoolchildren with dyslexia and those with ADHD showed very similar response times. Age was an important variable to be analyzed separately. As they aged, children with typical language development had fast answers on colors and digits tasks while children with dyslexia or ADHD did not show improvement with age.

**Conclusions:** The schoolchildren with dyslexia took longer to complete all tasks and ADHD took longer to complete digits and objects tasks in comparison to their peers with typical development. This ability tended to improve with age, which was not the case, however, with schoolchildren who had ADHD or dyslexia.

## Introduction

The Rapid Automatized Naming (RAN) test has its importance and relevance traced from nineteenth century (Denckla and Cutting, 1999) and albeit the task of naming familiar terms may seem simple at first, it helps to understand a complex circuit that are involved in fluent reading (Norton and Maryanne, 2012).

The effective development of reading and writing skills requires the concerted action of several abilities. Among these remarkable skills, phonological processing, which encompasses phonological awareness, rapid naming, and auditory memory, is reputed to be essential for reading and writing (Baddeley and Hitch, 1974; Wagner et al., 1994, 1997).

Rapid naming is the ability to identify and recognize a given item through the activation and concomitant articulation of its name, which will later be stored in the mental lexicon (Denckla and Rudel, 1974, 1976). The particular importance of rapid naming for the processing of reading and writing skills is well-established in the literature. In fact, ineffective rapid naming is known to be a marker of reading failure in preschool children.

Currently, the influence of phonological processing deficits as the key causative factor in reading and writing learning disorders is much discussed. Also, there are plenty of studies that have assessed the performance of individuals with attention deficit hyperactivity disorder (ADHD) compared with individuals with dyslexia and their typically developing peers, specifically with regard to their phonological processing abilities. However, RAN tends to be different in predicting dyslexia for each language. For example, all RAN tasks are reported to be slower for American children with dyslexia (Nicolson and Fawcett, 1994) while digit naming has a high predictive power for Chinese children with the same diagnoses (Shu et al., 2006). But, specifically in the Brazilian population, which constitutes the corpus investigated in this research, it is a theme little explored in depth, given the difficulty of access to multidisciplinary teams to establish differential diagnoses accurately.

Indeed, the major difficulty in conducting studies with these groups is related to diagnostic accuracy, since co-occurrences are common in these cases as well as the difficulties that could arise in establishing differential diagnoses. Nicolson and Fawcett (2007) reported a marked difficulty in distinguishing the “core” symptoms (specific and central to the disorder) from “secondary” symptoms (nonessential and, quite frequently, shared with other pathological conditions).

In studies concerning dyslexia and ADHD, the history of complaints and clinical presentation should receive particular attention and a thorough multidisciplinary assessment of the symptoms is required for a differential diagnosis. Recent studies have pointed to comorbidity rates ranging from 11 to 40% between these two conditions (Nicolson and Fawcett, 2007).

Poor academic performance and learning difficulties can occur in ADHD and thus compromise both reading and writing skills (Poeta and Rosa-Neto, 2004; Santos and Vasconcelos, 2010). The hallmarks of ADHD are inattention, hyperactivity, and impulsivity. Neurobiological and genetic aspects are primary etiologic factors of this disorder (Rohde et al., 2004; American Psychiatric Association, 2013), whose symptoms entail social, family, and school issues. It is important to note that executive functioning is impaired in individuals with ADHD. Consequently, the interaction between visual, attentional, linguistic, and auditory processing can be deficient, resulting in metalinguistic, and phonological difficulties for children. In some cases, this may compromise the acquisition of reading and writing skills (Ygual-Fernández et al., 2000; Oliveira et al., 2011; Cunha et al., 2013).

In addition, there are cases in which the usual modalities of learning are impaired since the early stages of development and these deficits are related to specific learning problems of reading and writing. This is the case of dyslexia, a specific language disorder of neurobiological origin characterized by difficulties in word decoding as a result of inefficient phonological processing (Lyon et al., 2003; Fawcett and Nicolson, 2007; Kamih and Catts, 2011). With regard to the etiology of dyslexia, the hypothesis of a phonological deficit has been accepted over the past few years. This hypothesis postulates that the difficulty in decoding written words is rooted in difficulties with the representation and/or access to phonological information (Fellipe and Colafêmina, 2002; Fawcett and Nicolson, 2007; Kamih and Catts, 2011).

The genetic-neurological disorders that are termed ADHD and dyslexia lead to academic failure (Poeta and Rosa-Neto, 2004; Rohde et al., 2004; Nicolson and Fawcett, 2007; Kamih and Catts, 2011; Cunha et al., 2013). Thus, it is of paramount importance to learn how the components of information processing are manifested in these two disorders (Nicolson and Fawcett, 2007). To measure the functioning of these components, RAN is one of the most important tasks that have been little explored in Brazilian literature.

The aim of the present study was to understand in Portuguese the differences in rapid automatized naming performance between schoolchildren with dyslexia and schoolchildren with ADHD and their unaffected peers, how each study group behaves in each sub-ability test, as well as understanding how the development of this skill happens with age.

## Materials and methods

The present study was approved by the institutional review boards of *Universidade Federal de Minas Gerais*—UFMG (Protocol No. CAAE05890203000-11) and *Centro Universitário Metodista Izabela Hendrix* (Protocol No. 214/2008). Both committees gave ethics clearance for conducting this research with humans.

### Participants

In total, 70 schoolchildren aged between 8 years 2 months and 11 years 1 month participated in the study: 40 children without complaints of language, hearing, or learning disabilities, 16 children with a multiprofessional diagnosis of ADHD, and 14 children with a multiprofessional diagnosis of dyslexia. All children in the study came from public schools in the same city. The ADHD group comprised 12 boys and 2 girls while the dyslexia group comprised 11 boys and 3 girls (Table 1). The sample size of children with dyslexia and ADHD can be justified for two reasons: 1. All children with any other language or communication disorders were excluded from the final data. Hence, from reading data for 42 children, the clinical group comprised 16 children after excluding all co-occurrences; 2. Brazilian multiprofessional centers for diagnosis are not common, which reduces the possibility of access for the diagnosis in some cities, particularly differential diagnosis.

**Table 1:** **Distribution of the study participants by gender**.

	**Control**	**Dyslexia**	**ADHD**
F	18	3	4
M	22	11	12
Total	40	14	16

*F, female; M, male; ADHD, attention deficit hyperactivity disorder*.

#### Selection of the control group sample

The control group population was recruited entirely from public schools, as were the schoolchildren in the ADHD and dyslexia groups. The same researcher collected the samples in both groups.

The children's parents or legal guardians were briefed about the study and received an informed consent form. Only the children whose parents or guardians agreed to their participation and provided written informed consent were included. In total, 137 completed informed consent forms were collected.

The allocation of the participants into groups with and without complaints of learning difficulties was performed using a questionnaire addressed to the children's parents or legal guardians (Lúcio and Pinheiro, 2013) and another addressed to the teachers (Lefèvre, 1972). These questionnaires made it possible to gather parental information concerning the children's potential difficulties, overall development, aspects related to speech and language learning, scholastic learning, and past medical history.

The questionnaire addressed to the teachers enabled an objective classification of the students in three categories: (a) students who read well, (b) students who read reasonably well, and (c) students who read poorly.

Based on that rating list, the children categorized as good readers were selected to compose the final sample of 40 students without complaints of oral and written language impairments.

The inclusion criteria were as follows: (1) the children should be regularly enrolled in a public school between second and fifth grade of elementary school; (2) written informed consent should be provided by the children's parents or legal guardians; and (3) the children should not have any health condition that could, directly or indirectly, affect the language development status as indicated by the questionnaire completed by the parents. Children were excluded if their parents or legal guardians did not provide written informed consent or if their parents or legal guardians reported the presence of any pathological condition unrelated to the language disabilities reported in the questionnaire.

Although the testing was performed in different locations (school and clinic), the researchers were careful to choose a silent and private room to test the control group.

#### Selection of the sample of the pathological groups (dyslexia and ADHD)

All 30 schoolchildren diagnosed with dyslexia and ADHD were given a multiprofessional clinical diagnosis by the team of *Laboratório de Estudo dos Transtornos de Aprendizagem—LETRA* (Learning Disorders Study Laboratory) of UFMG Hospital das Clínicas. All the children were evaluated by a neurologist, a psychologist, a speech-language pathologist, and audiologist, an occupational therapist, and an educationist, who jointly made the clinical diagnosis of dyslexia and ADHD based on the objective assessments conducted. It is worth emphasizing that none of these children had undergone any speech-language therapeutic intervention.

The assessments conducted by the team comprised a focused history, standard neurological examination (ENT), evolutional neurological examination (ENE) (Rotta, 1975; Wechsler, 2002), Chrysler Intelligence Scale for Children (WISC-III) (Sisto and Noronha, 2005), Bender visual-motor Gestalt test (Neto, 2002), motor evaluation handbook (EDM) (Beery and Beery, 2004), visual-motor integration test (VMI) (Bicalho and Alves, 2010), rapid automatized naming (RAN) test (Denckla and Rudel, 1976), semi-structured questionnaires, evaluation of memory, phonological, and orthographic processing, word decoding, fluency in text reading and comprehension, and mathematical reasoning evaluation.

The sample inclusion criteria were as follows: (1) children aged 8–11 years; (2) regularly enrolled in Minas Gerais public schools; (3) diagnosed with dyslexia or ADHD as verified by the LETRA team; (4) no co-occurring pathological condition of any origin; and (5) with written informed consent provided by the parents or legal guardians. Age was not treated as co-variant at first due to the small sample of participants on the clinical group. Children were excluded if they (1) failed to provide written informed consent signed by the parents or legal guardians; (2) were outside of the study age range, or (3) had a co-occurring pathological condition with dyslexia or ADHD.

### Measures

The RAN test (Denckla and Rudel, 1976) was the assessment measure. The test consisted of rapid “automatized” naming of pictured objects, colors, letters, and digits comprising four sets of stimuli printed in paper in a layout of five rows with 10 items each. The four sets of stimuli were: (1) colors (red, yellow, green, blue, black), (2) lower-case letters (a, d, o, s, p), (3) digits (2, 4, 6, 7, 9), and (4) pictures of everyday objects (umbrella, scissors, comb, clock, key). The stimuli in each set were presented in a randomized order totaling 10 times each. The examinees were instructed to name the visual stimuli as fast as they could. The time for naming (in seconds) was recorded with the aid of a stopwatch (Denckla and Rudel, 1974, 1976). The researchers calculated the mean times (in seconds) for naming each sub-item.

The RAN test was administered to the dyslexia and ADHD group participants by the LETRA speech-language therapists at the clinic on a pre-scheduled evaluation date. The control group participants were evaluated in their schools, at a time previously arranged with the teachers in order not to disturb the children's teaching-learning process.

### Sample composition

The participants were allocated into three groups: a control group of 40 schoolchildren without complaints of communication impairments; a group of 14 children diagnosed with dyslexia; and a group of 16 children diagnosed with ADHD.

The groups were also organized in a different arrangement in order to ensure that the experimental groups (EGs) had sufficient numbers of schoolchildren to allow the analysis by age ranges, considering that no statistically significant differences were found between the experimental groups. The allocation for the analysis by age ranges was as follows:

° CG1: schoolchildren in the control group aged 8–9 years;
° CG2: schoolchildren in the control group aged 10–11 years;
° EG1: schoolchildren with dyslexia or ADHD aged 8–9 years;
° EG2: schoolchildren with dyslexia or ADHD aged 10–11 years.

### Statistical treatment

Statistical analysis was performed with measures of descriptive statistics (mean, median, standard deviation, coefficient of variation, maximum, and minimum). Data were reviewed for normal distribution using Aderson-Darling statistics (*p* < 0.05) for all groups. To examine if dyslexia and ADHD could be analyzed together or independently to study age influences we conducted 2-sample *t*-test comparing directly children with dyslexia and ADHD. The matched children with vs. without the disorders were compared by two tailed student's *t*-test for independent samples (*p* < 0.05).

## Results

Means, standard deviations (SDs), and coefficients of variation were compared across groups. The performance of the groups in the RAN tasks is summarized in Table 2.

**Table 2:** **Descriptive statistics for control, dyslexia, and ADHD groups across RAN tasks**.

		**Median**	**SD**	**C. Var**.	**Min**	**Max**	**Range**	***N***
Control	Colors	41.9	5.10	12.19	28	50	22	40
	Letters	26.9	4.83	17.97	19	41	22	40
	Numbers	29.1	4.42	15.22	20	39	19	40
	Objects	56.9	7.98	14.03	41	75	34	40
Dyslexia	Colors	50.1	11.93	23.83	27	69	42	14
	Letters	38.9	17.65	45.41	17	80	63	14
	Numbers	36.1	12.28	33.97	17	60	43	14
	Objects	71.5	21.77	30.45	29	100	71	14
ADDH	Colors	49.4	14.71	29.76	26	73	47	16
	Letters	33.6	15.06	44.87	0	56	56	16
	Numbers	37.8	13.73	36.37	18	60	42	16
	Objects	69.1	18.48	26.75	35	103	68	16

*SD, standard deviation; C.Var., coefficient of variation; N, number of individuals tested*.

An overall comparison of the four groups showed that the letter-naming task was performed in less time than the other tasks by all groups, followed by the digit-naming task. The color and object-naming tasks took longer to be completed across groups.

**Figure 1:**
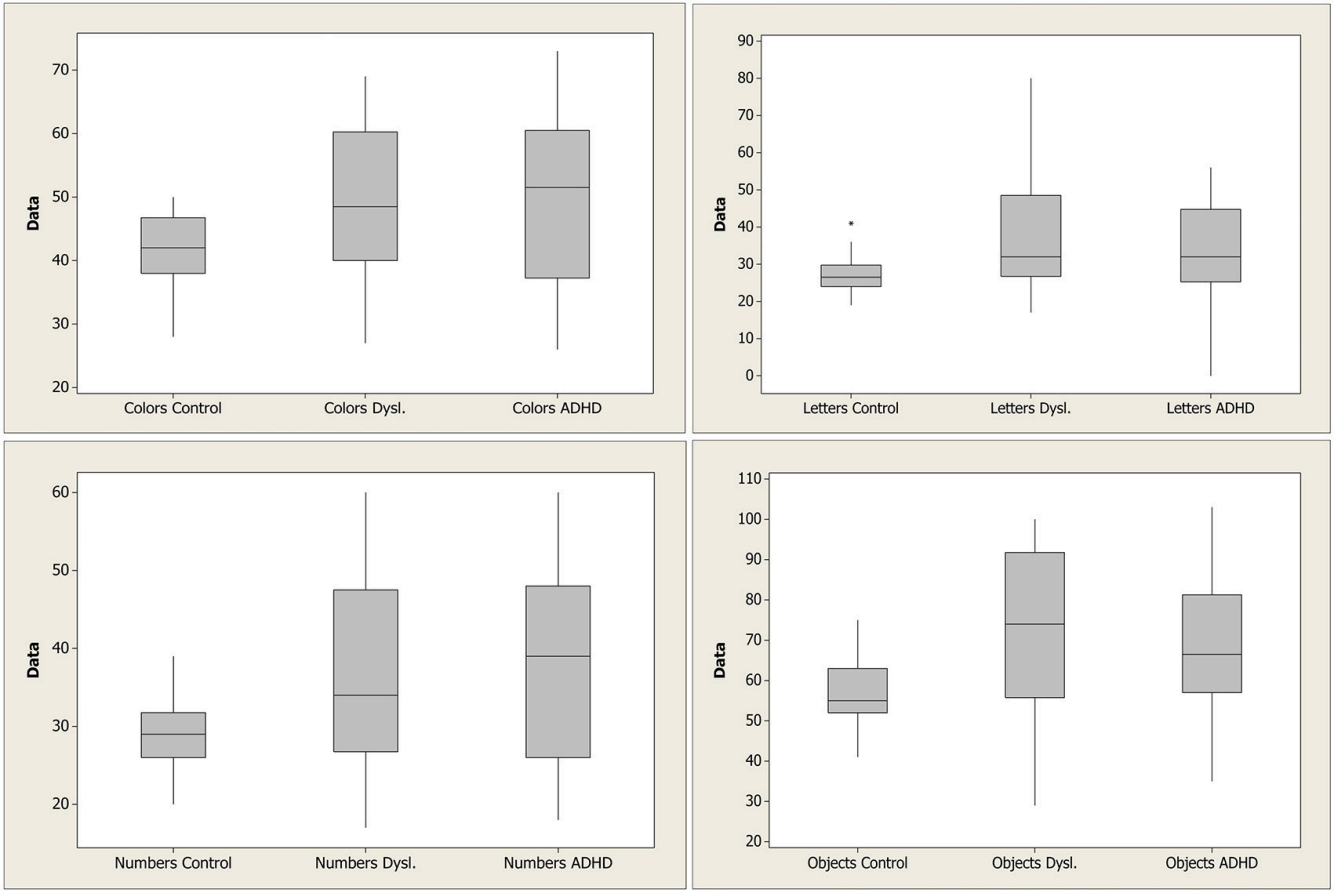
**First and third quartiles, medians, and boxplot outliers for the tasks of naming colors, letters, digits, and objects for the control, dyslexia, and ADHD groups**. ^*^, outliers; ADHD, attention-deficit/hyperactivity disorder.

**Figure 2:**
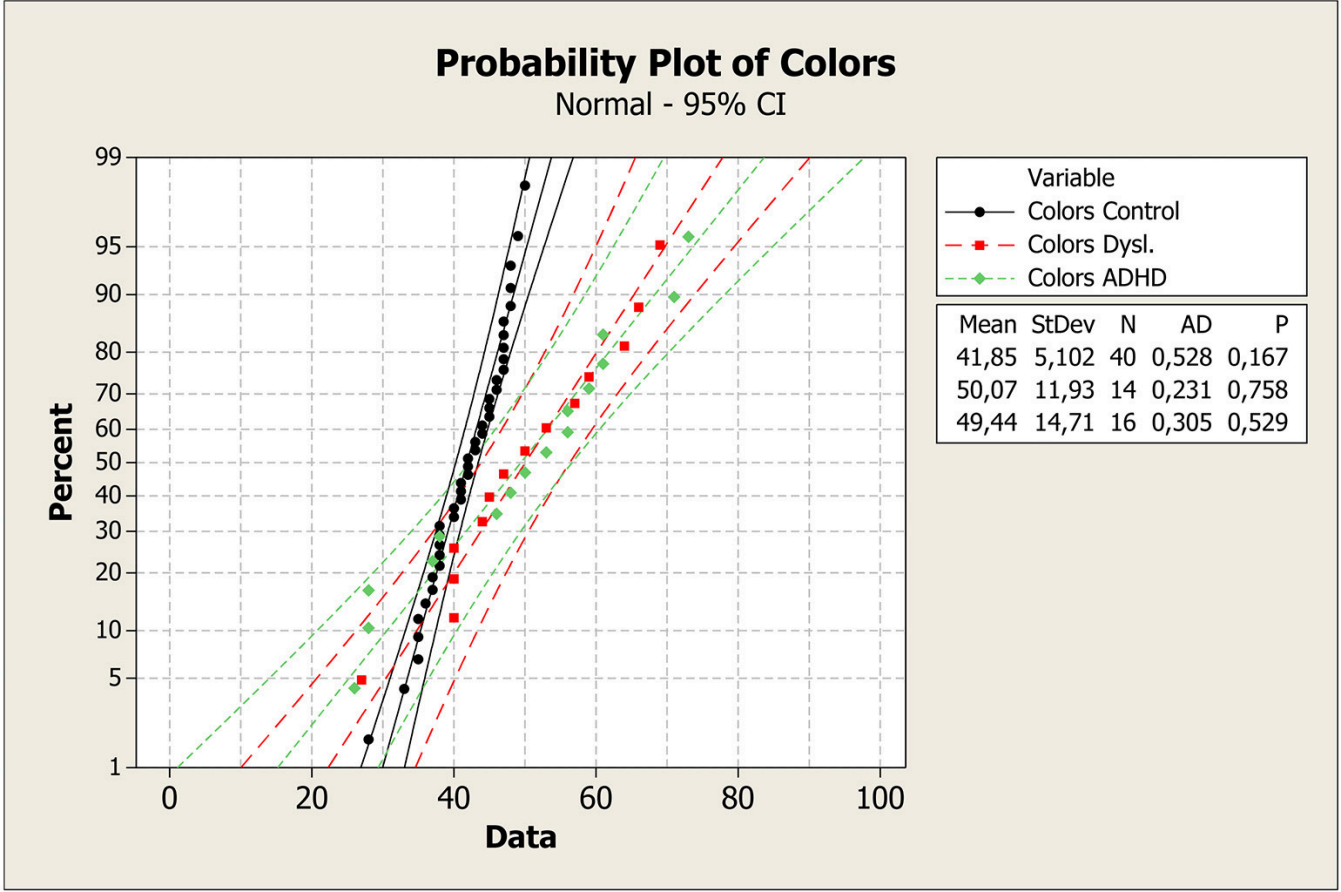
**Mean, standard deviation, ***p***-value, and probability plot for the RAN color-naming task for the control, dyslexia, and ADHD groups**. ADHD, attention-deficit/hyperactivity disorder; CI, confidence interval; StDev, standard deviation; N, number of individuals; AD, Anderson-Darling statistic; *p, p*-value.

**Figure 3:**
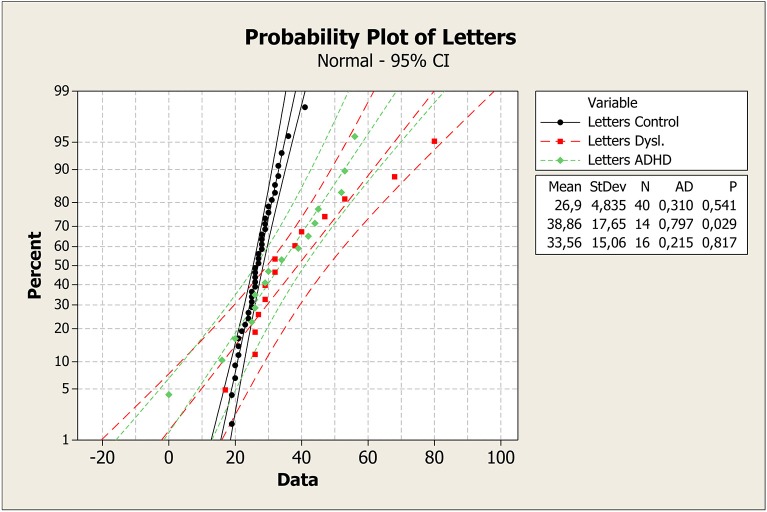
**Mean, standard deviation, ***p***-value, and probability plot for the RAN letter-naming task for the control, dyslexia, and ADHD groups**. ADHD, attention-deficit/hyperactivily disorder; CI, confidence interval; StDev, standard deviation; N, number of individuals; AD, Anderson-Darling statistic; *p, p*-value.

**Figure 4:**
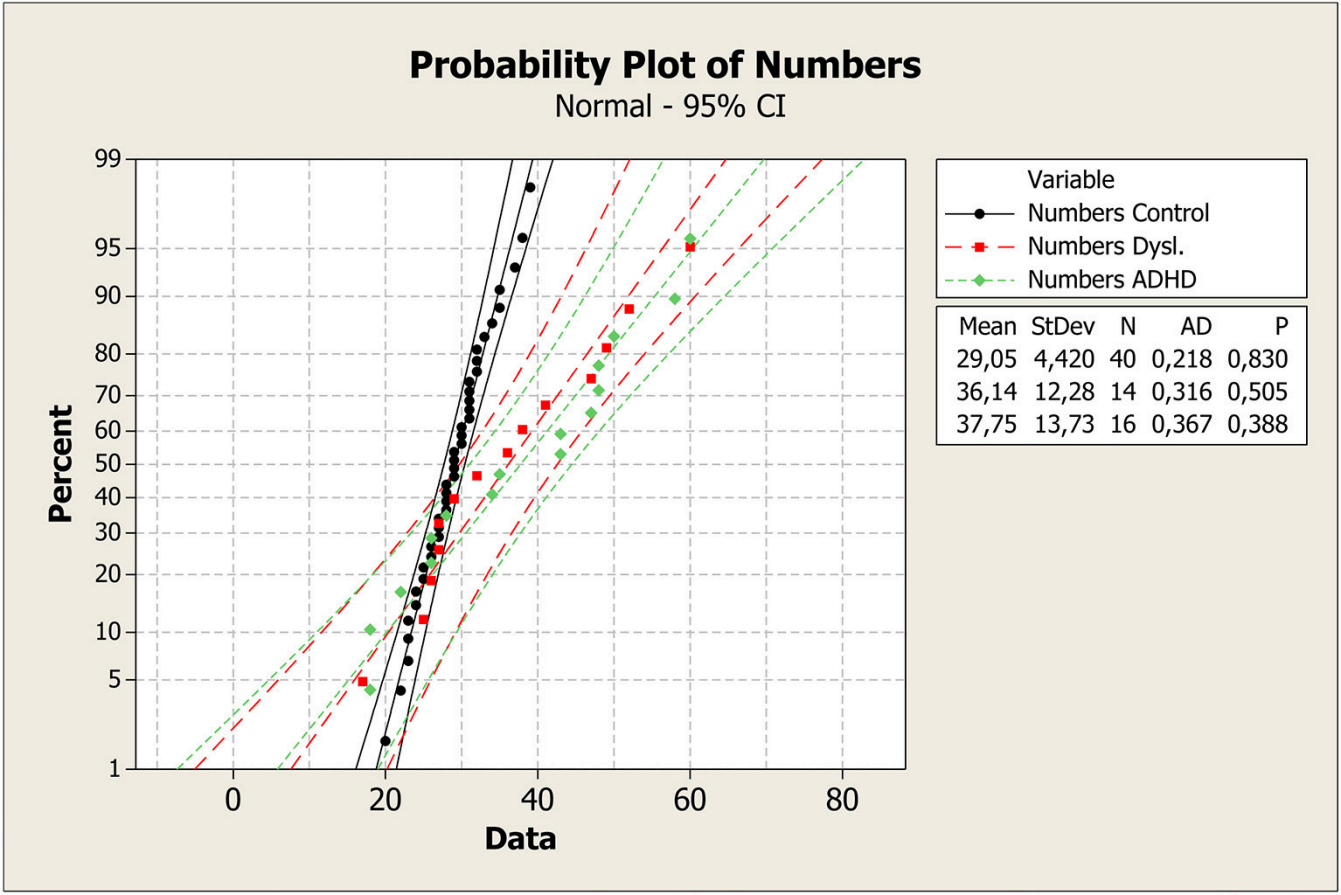
**Mean, standard deviation, ***p***-value, and probability plot for the RAN digit-naming task for the control, dyslexia, and ADHD groups**. ADHD, attention-deficit/hyperactivity disorder; CI, confidence interval; StDev, standard deviation; N, number of individuals; AD, Anderson-Darling statistic; *p, p*-value.

**Figure 5:**
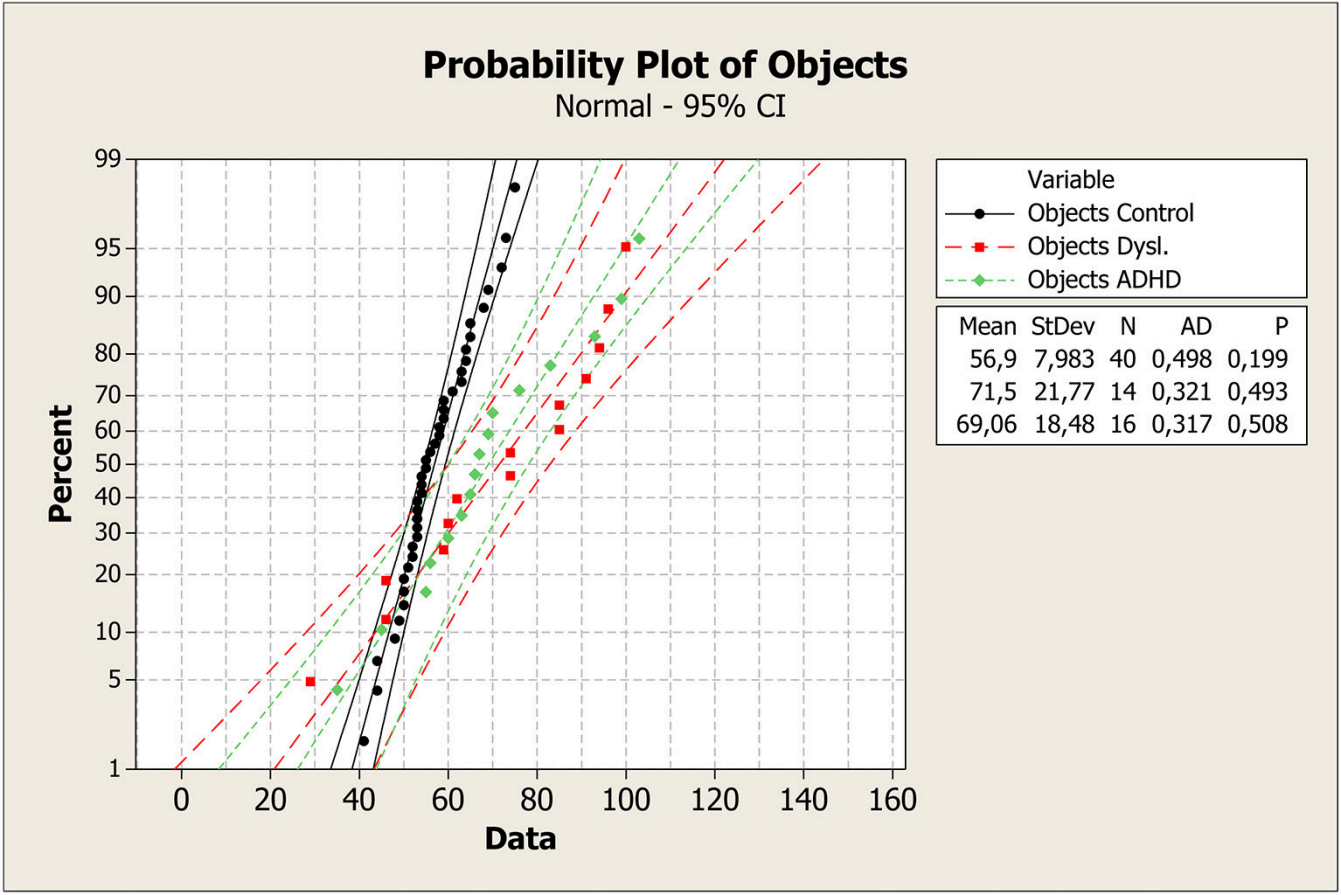
**Mean, standard deviation, ***p***-value, and probability plot for the RAN task of objects for control, dyslexia, and ADHD groups**. ADHD, attention-deficit/hyperactivity disorder; CI, confidence interval; StDev, standard deviation; N, number of individuals; AD, Anderson-Darling statistic; *p, p*-value.

To better understand these results, median values with the first and third quartile were measured in each task across groups, as shown in Figure 1. It is clear that control group had a faster response on all plots. Means, standard deviations, *p*-values, and probability plots for all RAN tasks are displayed in Figures 2–5 and illustrate the results described above.

Probability plot of colors shows that control group was faster in answering than dyslexia and ADHD groups (Figure 2). On the plot of Letters test, the same pattern can be seen (Figure 3). However, it is clear that dyslexia and ADHD are very close together, but ADHD is closer to the answer of the control group. For number and objects naming tests (Figures 4, 5), dyslexia and ADHD are still quite close, and ADHD group tend to deviate from control group.

Figures 1–5 show that: (a) all groups are normal distributed (Anderson-Darling statistics with *p*-value close to (1); (b) control group tend to be faster to respond RAN tasks; (c) dyslexia and ADHD have similar mean and, consequently, closer probability plot.

Therefore, the first comparison was due to determine if the matched children had indeed faster answers and the second comparison between groups was due to determine if dyslexia and ADHD could be studied together (or independently) to study age influence. To examine that it was conducted a 2-sample *t*-test comparing directly children with and without dyslexia (control vs. dyslexia), children with and without ADHD (control vs. ADHD), and children with dyslexia and ADHD (Table 3). With those results, it was decided to study children with dyslexia and ADHD in one group: experimental group (EG).

**Table 3:** **2-sample ***t***-test for between-group comparisons**.

	**Colors**	**Letters**	**Digits**	**Objects**
Control vs. Dyslexia	0.02^*^	0.03^*^	0.04^*^	0.03^*^
Control vs. ADHD	0.05	0.10	0.02^*^	0.02^*^
Dyslexia vs. ADHD	0.90	0.39	0.74	0.75

*ADHD, attention deficit hyperactivity disorder*.

* *Correlation is significant at the 0.05 level*.

When comparing the control group with the two pathological groups separately, significant differences were noted for nearly all sub-items tested, with the exception of the colors and letters and naming skill for ADHD. The comparison between the pathological groups yielded non-significant differences.

The means and SDs for the control and experimental (dyslexia or ADHD) groups separated by age range (8–9 years and 10–11 years) and the between-group comparisons are given in Table 4.

**Table 4:** **Mean, standard deviation, and 2-sample ***t***-test for comparisons between age groups**.

**RAN Task**	**Group**	**Mean**	**StDev**	**N**	***T*-value**	***p***	**Age-group**		***T*-value**	***p***
Colors	G1 control	47.1	4.2	18	3.2	0.0007^*^	G1	Control	2,53	0.03^*^
	G2 control	38.9	4.9	22				Exp.		
	G1 exp.	52.9	11.2	12	0.8	0.5	G2	Control	2.21	0.04^*^
	G2 exp.	48.1	14.8	8				Exp.		
Letters	G1 Control	28.4	5.2	18	1.9	0.07	G1	Control	2.36	0.03^*^
	G2 Control	25.7	4.3	22				Exp.		
	G1 exp.	37.6	12.7	12	−0.1	0.9	G2	Control	2.10	0.05
	G2 exp.	38.5	22.6	8				Exp.		
Digits	G1 Control	30.8	4.4	18	2.4	0.02^*^	G1	Control	2.31	0.04^*^
	G2 Control	27.6	4.0	22				Exp.		
	G1 exp.	39.3	13.3	12	0.9	0.4	G2	Control	2.51	0.02^*^
	G2 exp.	33.4	14.3	8				Exp.		
Objects	G1 Control	58.1	8.2	18	0.9	0.4	G1	Control	3.3	0.006^*^
	G2 Control	55.9	7.9	22				Exp.		
	G1 exp.	77.0	18.7	12	1.1	0.3	G2	Control	1.98	0.06
	G2 exp.	66.1	24.3	8				Exp.		

*SD, standard deviation*.

* *Correlation is significant at the 0.05 level*.

Age has shown to be an important variable to be analyzed separately. The mainly trends observed were: (a) as they aged, children with typical language development had faster answers on colors and digits tasks; (b) children with dyslexia or ADHD did not show improvement with age; (c) experimental and control group had statistic difference in all tasks for the younger groups; (d) experimental and control group had statistic differences in colors and digits tasks for the older groups.

**Figure 6:**
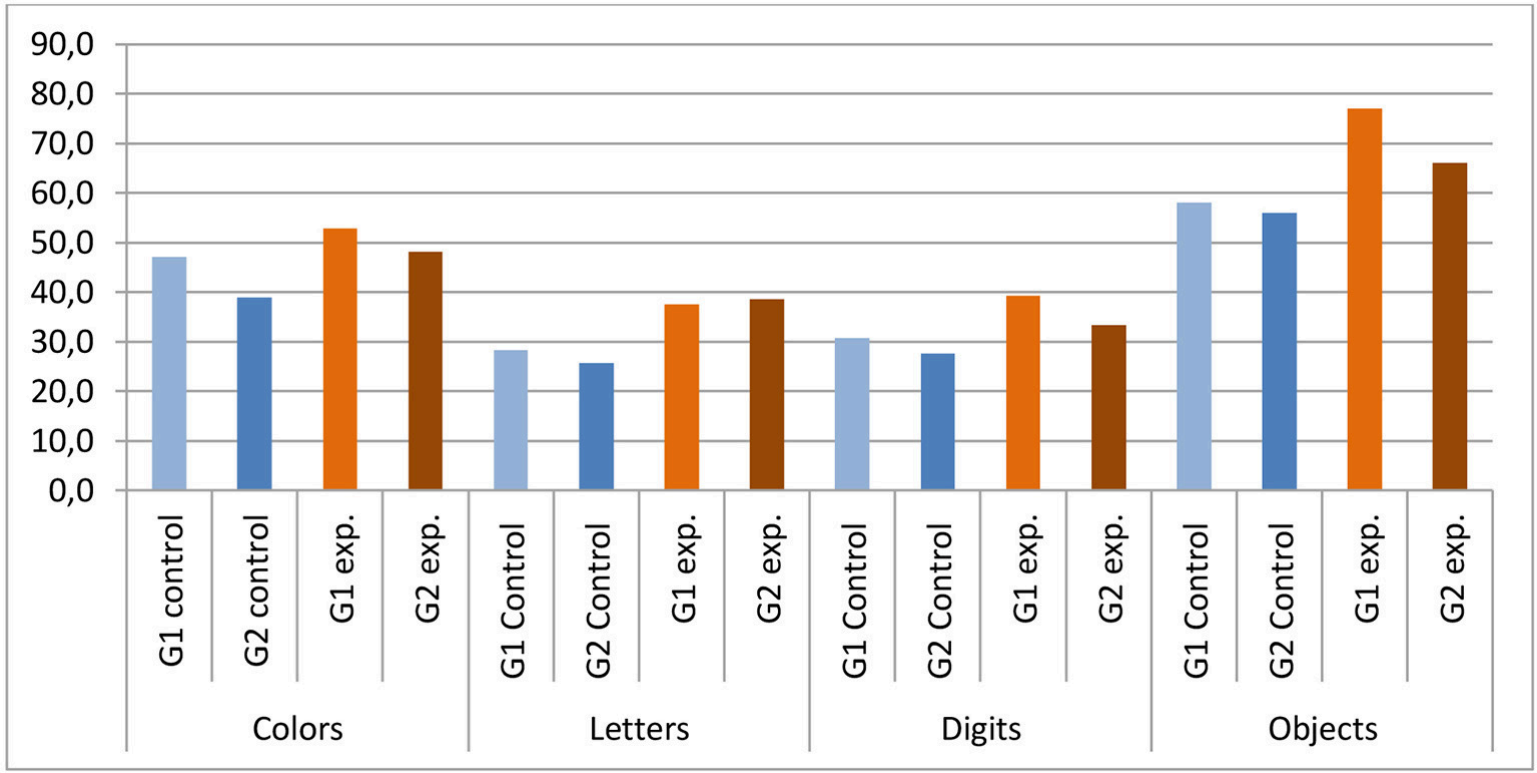
**Mean of the means for the control and experimental groups for each RAN task (colors, letters, digits, and objects)**. Note the differences in the performance of ranges 8–9 years (G1) and 10–11 years (G2).

Both in the control and the experimental group, there was a trend toward improved task performance, with naming becoming faster with age. However, this improvement is much more evident in the control group, with a statistically significant difference between age ranges for color and digits tasks. By contrast, this difference, albeit observed in Figure 6, is not statistically significant for the experimental group age ranges, as shown in Table 4.

## Discussion

The objective of the present study was to determine whether there is a significant difference in RAN performance between schoolchildren with ADHD and with dyslexia compared with their peers with no reading and writing development deficits, as well as understanding how the development of this skill happens with age and what are the main predictors of developmental disorders.

All groups performed the letter and digit naming tasks faster and easier than they did with colors and objects. The naming of objects was shown to be significantly slower, followed by colors, which corroborates studies previously conducted (Pennala et al., 2010; Whipple and Nelson, 2015). According these studies, the above results can be explained by the fact that naming pictures always requires access to their meaning for subsequent articulation of their name. Letter-reading, on the other hand, can bypass this process, since the identification of a grapheme or digit does not require access to meaning. Objects and colors involve a greater semantic load, which justifies the greater length of time needed for naming these items (Denckla and Rudel, 1974; Whipple and Nelson, 2015). From a neurobiological point of view, naming objects and colors tasks require a higher working memory demand, both visuospatial as verbal and semantics.

Literature points to a phonological deficit as the causal factor of the impairments in written language (Ygual-Fernández et al., 2000; Fellipe and Colafêmina, 2002; Lyon et al., 2003; Fawcett and Nicolson, 2007; Araújo et al., 2011; Kamih and Catts, 2011; Cunha et al., 2013). The present study was intended to determine whether, among the different skills involved in phonological processing, RAN would be impaired in individuals with ADHD and dyslexia in different ways. The first finding was that the group of children with dyslexia had significantly worse performance for all tested tasks. This finding demonstrated that the rapid naming is a strong predictor of dyslexia. This finding is reported in the literature surveyed in different languages (Capellini et al., 2007; Krasowicz-Kupis et al., 2009; Fleury and Avila, 2015; Liao et al., 2015), although not in the same manner (e.g., digit naming has a higher predictive power for Chinese (Shu et al., 2006).

The results of the intergroup comparisons showed that both groups of schoolchildren with dyslexia and with ADHD performed worse in the tasks of naming digits and objects, with statistically higher means than those of the CG, indicating that those children took longer to complete the tasks. These outcomes corroborate the findings of others studies, which concluded that schoolchildren with ADHD and those with dyslexia have poorer performance in the RAN test when compared with their peers without learning disabilities (Purvis and Tannock, 2000; Raberger and Wimmer, 2003; Germano and Capellini, 2008; Gooch et al., 2011). These results indicate deficits in information processing, i.e., phonological processing in its component skill of rapid access to the lexicon.

However, that difference in performance was not found in the colors and letters naming task for children with ADHD. In other words, they have proven to be less committed on rapid naming skills in general. Interestingly, no significant difference was found in the RAN task performance between the schoolchildren with dyslexia and ADHD. This was previously mentioned in a 2007 study, which indicated that individuals with dyslexia are more deficient in RAN skills than those with ADHD (Raberger and Wimmer, 2003).

In line with the present study, several other studies have also demonstrated impaired phonological processing in children with dyslexia and ADHD corroborated by the outcomes of tests for phonological awareness, rapid naming, and auditory memory skills (Capovilla and Capovilla, 2002; Raberger and Wimmer, 2003; Souza et al., 2007; Tiedemann and Messina, 2009; Araújo et al., 2011; Oliveira et al., 2011; Jong et al., 2012; Cunha et al., 2013; Granzotti et al., 2013; Ramus et al., 2013).

The data obtained for the participants with dyslexia, specifically, lead to the reflection that the process of reading development is associated with the processing speed of visual information. In light of this, and considering agility in recognizing printed items a crucial factor for reading, it was already expected, based on theoretical assumptions, that the performance of the dyslexia group would be inferior to that of the control group (Denckla and Rudel, 1974; Jong et al., 2012).

On the other hand, the rationale for the similarly inferior outcomes of the ADHD group is that phonological processing is related to attention and to working memory, which is a neuropsychological function connected to other functions, responsible for selecting vital information and filtering out less relevant stimuli (Li et al., 2009). These children experienced, therefore, greater difficulty with phonological processing, with symptoms that could reflect in the performance of reading and writing tasks. The impairment in rapid naming is not specific of reading disorders; in fact, recent studies support the findings of the present study by showing a relationship with rapid naming deficits in individuals with ADHD, chiefly in the rapid reading of letters, colors, and objects (Tenório and Ávila, 2012).

In addition, it should be noted that those tasks are closely related to other components of phonological processing, such as auditory memory—without overlooking the fact that, in the case of the RAN test, the tasks engage resources of the subordinate systems of the phonological loop, and directed to passive phonological storage. Thus, the tasks assessed in the present study are linked to other skills pertaining to phonological processing. Further studies are warranted to investigate the other skills in an integrated, inter-related approach (Baddeley and Hitch, 1974; Jong et al., 2012).

One of the results of the present study came from the comparison between age groups. Some studies indicate that the ability of lexical access and working memory show no significant trend, especially between the ages of 7 and 8 years among children with typical development (Cardoso-Martins and Pennington, 2001; Norton and Wolf, 2012). However, our findings point toward a different direction: the participants with typical development showed a clear improvement in their rapid-naming skills, as evidenced by the statistically significant difference between groups 8–9 and 10–11 years. This trend was not observed significantly in the experimental group, which implies that schoolchildren with ADHD and dyslexia do not develop this skill adequately. This finding supports the hypothesis of a phonological processing deficit in individuals with dyslexia, and also indicates the difficulty of schoolchildren with ADHD in processing the rapid naming sub-component of phonological processing.

Further, the analyses by age range indicated statistic difference when comparing younger and older children in the control group for colors and digits naming tasks. That shows that children with typical development evolve those abilities with neurobiological maturation. A recent study has shown that, when children begin the literacy process, the practice and exposure to alphanumeric stimuli increase, which contributes to the automatization of the naming process (Purvis and Tannock, 2000). However, this development is not followed by individuals with dyslexia and ADHD. It is widely known the relationship between the ability of rapid serial naming performance in reading and writing, as well as the difficulties of children with dyslexia and ADHD in this ability (Purvis and Tannock, 2000; Souza et al., 2007; Tenório and Ávila, 2012; Germano and Capellini, 2008; Gooch et al., 2011). The investigations about the rapid naming testing has had a long history since Denkla and Rudel, but interesting aspects must still be observed in the studies in this area, as driving significant tests for different populations, either by age or by diagnostic specification submitted by each school. Our findings emphasize the importance of focusing on specific results for the task of objects and digits in younger children, which proved to be an important predictor of impairment and growing by comparing the groups. This is because the digit-naming test was the one that showed a statistically significant difference to the comparison between both groups by age and by pathological picture.

Considering that the present study involved a small sample, it is suggested that further studies should be conducted with larger populations. In addition, an investigation of the other skills related to phonological processing should be undertaken to determine the interplay of these skills and how they influence the process of learning to read and write.

Schoolchildren with ADHD or dyslexia took longer to perform tasks of rapid naming digit and objects compared with their typically developing peers. This deficit compromises the relationship between the skills of naming and automatization of stimuli, both necessary for the development of reading and writing. The rapid-naming skill improves with age in schoolchildren with typical development, which does not occur with schoolchildren who have ADHD or dyslexia, due to the neurobiological changes present in these frames.

Finally, some limitations of this study must be pointed out. The first point to be raised is that the RAN test was conducted with participants who were selected on the basis of an assessment that included RAN test results. Thus, it could not expect a predictor effect of the test, in this study, since it would obviously indicate a positive effect, but it does aid to understand, in Portuguese, the behavior of each group studied in the sub-skills tested as well as the effect of age in each group. It is also important to highlight that, considering the fact that the data recording was conducted in different contexts according to the groups (school for students of the control group and clinic for the experimental groups), we assume that the testing environments could have affected the results.

### Conflict of interest statement

The authors declare that the research was conducted in the absence of any commercial or financial relationships that could be construed as a potential conflict of interest.
